# The Position of Surgery in the Treatment of Septic Peritonitis1Read by invitation at the fortieth annual meeting of the Medical Association of Central New York, held at Rochester, October 15, 1907

**Published:** 1908-02

**Authors:** J. Wesley Bovée

**Affiliations:** Professor of Gynecology, George Washington University, Washington, D. C.


### The Position of Surgery in the Treatment of Septic Peritonitis.[1]

By J. WESLEY BOVÉE, M. D.,

Professor of Gynecology, George Washington University, Washington, D. C.

WHEN I was honored by an invitation to read a paper on this subject before this association I was led to believe the paper to be read by Dr. Stockton would deal with the non-surgical treatment of septic peritonitis; hence, the title of this paper. One might infer from various discussions on this subject that surgeons are earnest advocates of the non-surgical treatment of this disease or complication of other diseases. The discussion of Dr. Hotchkiss's paper, read at the meeting of the New York Surgical Society, April 11, 1906 (*Annals of Surgery*, 1906, XLIV 197-208), illustrated the divergent views of the surgeons present. The same may be stated regarding the dis-

1. Read by invitation at the fortieth annual meeting of the Medical Association of Central New York, held at Rochester, October 15, 1907

cussion of another very interesting paper read by Dr. J. G. Sherrill, of Louisville, at the meeting of The Southern Surgical and Gynecological Association in 1904 (vide Transactions Southern Surgical and Gynecological Association, 1904). Both were discussed by the ablest abdominal surgeons of this country.

To repeat, one might infer they regarded the treatment as non-surgical since each speaker deplored the lack of success attending his operations, while some vigorously condemned the technic of others in their operations. I can not in this paper do more than to direct your attention to the indefinite character of the term "septic peritonitis," to the many varieties thereof, to the treatment of them briefly, varying as it must because of the great number of conditions causing septic peritonitis, the necessity of treating the underlying cause and the intensity of the invasion, together with the comparative amount of area of peritoneum involved.

By first stating the conditions under which septic peritonitis may be, in my judgment, best treated medically or without operation, I may more clearly reach the much larger class,—the surgical one. In cases in which the invading organism is the streptococcus and the area involved large, I much doubt the advisability of opening the abdomen as death is almost sure to result. In such, stimulation and injections of anti-streptococcic serum are the main elements in treatment.

Even when the area is small, surgical treatment is questionable. Were the streptococcus accompanied by the colon bacillus, the gonococcus, the staphylococcus or even the gas bacillus, I would regard it as a surgical condition. There can be no question that oftentimes localised septic peritonitis from the gonococcus, the colon baçillus or the staphylococcus is successfully combated by nature or is combated for a sufficient amount of time for death of the invading organism to occur, when a secondary and much safer surgical operation may be needed to eradicate the disease. There is, likewise, no doubt that such localised invasions are at times entirely overcome by the processes of nature. Probably the larger part of such instances are those in which the portal of invasion has been the Fallopian tube with the peritoneal involvement distinctly localised in the pelvis.

We also find, accidentally, that perforation of an infected gall-bladder, of the intestine, the oviduct and even the stomach has occurred producing peritonitis, local in character, and reparative processes have overcome the difficulty. I mention these latter more as possibilities than results typical or to be expected from treatment. Whether a non-surgical plan of procedure is the proper one in cases of advanced septic peritonitis sequent to perforations of hollow viscera, in which a fatal termination seems almost inevitable, is one always to be determined in each individual case and largely by the personal equation of the attending surgeon,—the pendulum swinging on one side toward doing everything possible for the good of the patient, and on the other to conserving personal statistics and upholding the popularity of surgery in the community. Between these two the surgeon should never hesitate.

The brilliancy and popularity of surgery should never be gained by surgical cowardice. The welfare of the patient in each individual case is the only healthy motto in such work. The personal characteristics of the individual surgeon have more influence in molding public disregard for surgery than surgical bravery in cases regarded as nearly moribund. I would, therefore, limit non-surgical treatment in this class to those cases that have passed beyond consciousness, and in which the absorbents have been permanently suspended with death imminent. Mention here might be made of other complications, such as hopeless lesions of vital organs as complicating septic peritonitis. In this class might be found exaggerated cases in which surgical intervention would be interdicted.

Having thus mentioned a few conditions in which non-surgical treatment of septic peritonitis would be not only proper but preferable, I will at once state it as my opinion that all other classes or phases of peritonitis of a septic character should be treated surgically.

There can be no doubt, in my judgment, that in perforations of the stomach, gall-bladder, intestine and pancreas subsequent to infectious processes that peritonitis is rapid and severe in type. Of course, much depends upon the virulence and number of the organisms present and upon the resistance of the individual attacked. Gunshot wounds, particularly from bullets of small caliber, is least harmful of this class. This is due to the normal condition of the tissues involved. Many such openings in the intestine collapse, particularly if the viscus be not distended at the time of injury, and but slight infection occurs, which is successfully combated by the resistive powers of the body. This fact was well attested in the late war between this country and Spain and that between Russia and Japan. Even injuries to the ureters or bladder with leakage of normal urine in small quantities into the peritoneal cavity occur without severe peritonitis. But perforations of the stomach and pancreas permit escape into the peritoneal cavity of highly irritative substances that promptly lead to septic peritonitis. Perforations of the intestine sufficiently large to permit fecal extravasation of notable amount, may always be relied upon to produce septic peritonitis of a general character. The same may be said of the escape of material from the bile tract. In perforation of the vermiform appendix during the process of virulent appendicitis, or of a periappendiceal abscess containing virulent organisms, we must expect the same result to follow with great rapidity. When rupture of such abdominal tumors as suppurating or infected ovarian dermoid cysts or uterine fibroids occurs the rapidly following septic peritonitis must always be expected.

In all these conditions prompt abdominal section offers by far the greatest possibility of relieving the condition. An essential feature of the treatment, if early operation can be done, is careful closure of the visceral perforation or removal of the tumor, if such be present. Special care, of course, is necessary when necrotic areas about the opening exist, as is present in appendicitis and typhoid perforations.

Several points in technic and after-care are yet to be decided as various opinions exist. These, together with the high mortality rate, are what caused in the discussions mentioned a possible inference that surgeons believed in the non-surgical treatment, which, in fact, is not true. These points are:

1. The amount of time to which the patient is to be subjected to operation.

2. The variety of anesthetic to be used and the degree of anesthesia.

3. The amount of manipulation of the abdominal contents.

4. The use of salt solution for flushing out the peritoneal cavity and wiping carefully the peritoneum with gauze.

5. Drainage: postural and by materials.

6. The use of foods after operation.

7. The after-treatment of the gastro-intestinal tract.

8. The postoperative administration of morphia.

Patients, whose power of resistance is so lessened as in septic peritonitis plainly should not be required to withstand the enormous taxation of a prolonged operation. Whatever is to be done must absolutely exclude an operation requiring more than a few minutes. The more advanced the stage of inflammation, or the more intense the infection, the more vital becomes this fact. A quick operative procedure may save several borderline cases while a slow operation might remove all hope of recovery.

As ether and chloroform anesthesias are depressing to vital forces these should not, as a rule, be employed to the extent of profound narcosis, and when employed should be administered by an expert whenever it is possible to secure such services.

Local anesthesia has a strong indication in this work, and I have a few times successfully opened the abdomen in this condition without administering any sort of anesthetic. To those who are accustomed to the use of morphia in such conditions I would recommend the employment, sparingly, of the hyoscyaminmorphia form of anesthesia. In advanced cases the toxemia present may obtund the sensibilities to the extent of rendering unconsciousness unnecessary.

The amount of manipulation of the abdominal contents is a very important matter. It is to be avoided as much as possible. To get into the abdomen as quickly as possible and out again as soon as possible, quoting from John B. Murphy and a few other prominent surgeons, is a splendid rule. Yet it would be only in extreme cases that I would follow the plan of some, of merely opening the abdomen and allowing material to escape, relying principally upon the relief of tension to do practically all for the patient. In cases nearly moribund, however, this is all that is justifiable and local anesthesia, or none at all, is all that should be employed. Unquestionably, manipulation of the viscera in such cases disturbs the epithelium permitting more rapid absorption of toxins, as well as pathogenic organisms, and greatly shocks the patient.

The use of salt solution for washing out the peritoneal cavity is, in my judgment, of doubtful value. I believe it, as a rule, is harmful notwithstanding it has earnest advocates. The conscensus of opinion is that it can be done too much; that shock results from extending it beyond five minutes, and that it does tend to interfere with nature's regulation of cell activity of the peritoneal epithelium. Moreover, it tends to carry septic material to healthy areas should such areas remain. That it overtaxes the absorptive power of the lymphatic system, which oftentimes is crippled, can not be gainsaid. It can not do as much as postural drainage in such conditions. The practice of wiping loops of intestine, with or without evisceration, has not proven satisfactory. I was glad to hear its former strong advocate, Finney, of Baltimore, declare his regret for his advocacy of this plan and that he had abandoned it. It has great opportunities for evil.

The question of drainage is still perplexing. Many advocate it and perhaps as many express a determination to drain less. Much depends upon the toxemia present and the general condition of the patient. If good, more reliance can be placed upon nature to control the situation. In the presence of much necrotic material or, in the case of perforations we have doubt of perfect closure, drainage had best be instituted. When the disease is localised and it can be satisfactorily dealt with by the surgeon at the time of operation, drainage may be dispensed with to advantage. In all other conditions I feel it is safer to drain. Here position is of importance and when the area of infection is large, openings in the lower part of the abdominal wall and the patient in nearly a sitting position are decidedly advantageous. Gauze alone is not good drainage material, but with a rubber tube or rubber tissue, in the form of a cigarette drain, it is probably the best we have. The gauze should always be rolled loosely, as if tight it obstructs drainage. Nor should it protrude far through the rubber material in the peritoneal cavity or be tightly constricted in its passage through the abdominal wall. It should be remembered the drainage power of such a drain depends entirely upon the diameter of it at the point of constriction by the abdominal wall.

The use of foods after operation, while of considerable importance, should not be a subject for wide variance of opinion. I am well aware that prominent internists differ even as to the diet in typhoid fever, some thinking a milk diet best, others that liquid meat preparations are superior, while yet others would prefer rare meats. But in this disease one must be guided largely by the area involved, the stage of the disease and above all the cause or forerunner of it. I believe no surgeon can direct the diet of a case after operation unless he is personally familiar with the conditions confronting him.

The after-treatment of the gastro-intestinal tract is of special moment and I would suggest that for the first three days no effort to produce bowel movements should be made if no distension be present. We should await natural conduct of this tract. If at the end of that period of time the peristaltic action has not been sufficient it may be stimulated by simple or purgative enemata, gentle laxatives by the mouth, or the hypodermic administration of salicylate of eserine, as so strongly urged by Craig, of Boston. Should intestinal distension be present, I would think, until the tension is reduced and a degree of comfort ensues, brisk catharsis is indicated. Two exceptions to this would be preceding intestinal obstruction particularly if cathartics have been freely employed unsuccessfully, and if extreme weakness is a feature of the condition. In the one case we have to guard against the free action of the previously administered cathartics and in the other against over exhaustion.

I am not a believer in the Alonzo Clark treatment of peritonitis by opium splints. As opium has a strong tendency to lessen all cellular activity, I prefer to withhold it entirely, if possible, relying upon the coldwater coil or ice as an analgesic and stimulating, if possible, the emunctories by the use of salt solution by hypodermoclysis or bowel,—or even intravenously if necessary, and by considerable quantities of fluids by the mouth, when admissible.

No matter what the treatment has been the records show that this is a highly fatal malady. So far as I know no man has had an equal success with Murphy, of Chicago, and we may yet change entirely to his plan of relieving tension and trusting to nature for the rest.

"THE ROCHAMBEAU," 815 CONNECTICUT AVENUE.

